# Optic Neuritis in the Older Chinese Population: A 5-Year Follow-Up Study

**DOI:** 10.1155/2017/3458356

**Published:** 2017-12-11

**Authors:** Junqing Wang, Huanfen Zhou, Limin Qin, Chunxia Peng, Jie Zhao, Da Teng, Dahe Lin, Nanping Ai, Quangang Xu, Shihui Wei

**Affiliations:** ^1^Department of Ophthalmology, Chinese PLA General Hospital, Beijing, China; ^2^Department of Ophthalmology, The First Affiliated Hospital of Chinese People's Liberation Army General Hospital, Beijing, China; ^3^Department of Neurology, Chinese PLA General Hospital, Beijing, China

## Abstract

**Objective:**

This study aims to describe the clinical manifestations and outcomes in a cohort of older Chinese patients.

**Method:**

A retrospective study of patients aged ≥ 45 years who had a first episode of optic neuritis (ON) between May 2008 and November 2012. Clinical features at onset and last follow-up were analyzed within subgroups (age 45–65 years and age ≥ 65 years).

**Results:**

76 patients (99 eyes) were included, of which 58% were females. The mean age at presentation was 55.53 ± 8.29 years (range: 45–83 years). Vision loss was severe at presentation, with initial best corrected vision activity (BCVA) < 20/200 in 93% and final BCVA < 20/200 in 53% of patients at 5-year follow-up. Final BCVA significantly correlated with the initial BCVA and peripapillary retinal nerve fiber layer. At last follow-up, 14.5% were diagnosed with neuromyelitis optica spectrum disorder (NMOSD), 1.3% were diagnosed with multiple sclerosis (MS), 5.2% with chronic relapsing inflammatory optic neuropathy, 1.3% with infectious ON, and 19.7% with autoimmune ON. None of the elderly group (≥65 years) developed NMOSD or MS.

**Conclusion:**

Chinese patients in the age group ≥ 65 years with ON are less likely to develop NMOSD or MS. Notwithstanding, they had more severe visual loss at onset and poor recovery.

## 1. Introduction

Optic neuritis (ON) is the most common inflammatory demyelinating neuropathy [1]. It can manifest as either an isolated episode or be caused as a result of the patient suffering from multiple sclerosis (MS) or neuromyelitis optica spectrum disorders (NMOSD) [2, 3].

The optic neuritis treatment trial (ONTT) studied patients between 18 and 45 years old with acute, unilateral optic neuritis; most of whom had good vision recovery [4]. This has been classified as typical ON [5]. In ON patients, ethnicity has been documented as an important factor influencing clinical features and the outcome [6]. It has been identified that NMOSD is more likely to develop in Asia, whereas MS is more prevalent in western countries [7]. Previously, we reported a cohort of Chinese ON patients with atypical clinical features and poor recovery [8]. African-American patients were also found to have more severe vision loss and less vision recovery [9].

Although, the ONTT is the largest study to date of ON; it included only patients between 18 and 45 years. There are, currently, only a limited number of studies on the clinical features of ON in the older population. We previously reported a cohort of Chinese ON patients of all ages [10]. We observed less vision recovery in patients older than 45 years, compared to patients younger than 45 years. However, we could not divide patients into more specific age groups for analysis due to the small number of patients. We therefore carried out this study to describe the clinical manifestations and investigative outcomes in two age groups, middle-aged patients (45–65 years) and elderly patients (>65 years).

## 2. Patients and Methods

This retrospective study included patients evaluated for a first episode of optic neuritis in the Neuro-ophthalmology Clinic of the Chinese PLA General Hospital (PLAGH) in Beijing between May 2008 and November 2012. Only patients who had a first episode of ON were included in the study. To compare clinical features, we divided patients into two groups, middle-aged group (45–65 years) and elderly group (older than 65 years).

Inclusion criteria are the following: (i) the first episode ON with BCVA loss; (ii) older than 45 years; (iii) follow-up of ≥5 years; (iv) no prior steroid treatment; (v) objective evidence of visual abnormalities: relative afferent papillary defect (RAPD), visual field defects, and abnormal visual evoke potential (VEP).

Exclusion criteria are the following: (i) nonarteritic anterior ischemic optic neuropathy (NAION), (ii) disorders other than ON, including compressive, hereditary, vascular, toxic, traumatic, metabolic, or infiltrative neuropathy, (iii) ocular comorbidity including uveitis or glaucoma, and (iv) amblyopia.

To exclude a diagnosis of NAION, known risk factors for ischemic disease were evaluated, including [11] (i) systematic conditions: hypertension, diabetes, hyperlipidaemia, sleep apnoea syndrome, and nocturnal hypotension; (ii) medical history: erectile dysfunction drugs, amiodarone, and cataract surgery; (iii) “crowded” optic disc.

The protocol of the study was approved by the institutional review board at the Chinese PLAGH and performed in accordance with the tenets of the Helsinki Declaration. Informed consent was obtained from all the patients.

### 2.1. Clinical, Immunological, and Radiological Assessment

All patients underwent the ophthalmic exam, including ocular symptoms, best corrected visual acuity (BCVA), pupil function test, optic disc appearance, colour vision test, optical coherence tomography (OCT), and fluorescence angiography (FFA). The BCVA in all patients was evaluated using the Snellen visual chart, and the data was transformed as the logarithm of the minimum angle of resolution (logMAR) values [12]. In patients with bilateral optic neuritis, only the eyes presenting the worst BCVA at onset were included in this study.

Because of the retrospective nature of the study, some investigation results were unavailable for a number of patients. Orbital magnetic resonance imaging (MRI) and head MRI were used to detect the Gd-enhancement in the optic nerve or in any brain lesion. The MR images were reviewed by the same experienced radiologist to ensure consistency of the results. Serums were screened for antinuclear antibody (ANA), antineutrophil cytoplasmic antibodies (ANCA), extractable nuclear antigen antibodies (SSA and SSB), anticardiolipin antibodies (ACLs and *β*2-GPI), rheumatoid factor (RF) and major histocompatibility complex-B27 (HLA-B27), and aquaporin-4 antibody (AQP4-Ab) status. The serum AQP4-Ab level was assessed using the cell-based assay [13].

### 2.2. Statistical Analyses

All statistical analyses were performed using SPSS 22.0 software (IBM Corporation, New York, USA). Chi-squared Pearson test was applied for categorical variables and Mann–Whitney *U* test for quantitative variables. The Pearson correlation test was also used to determine the relationships between the recovery indices. A value of *p* < 0.05 was considered statistically significant.

## 3. Results

### 3.1. Demographic and Clinical Characteristics

76 patients (99 eyes) were included in this study, of which 44 were females (57.9%). The mean age at onset was 55.5 ± 8.3 (range: 45–83) years. The follow-up duration ranged from 5–8 years, with a mean of 5 ± 1.49 years. There were 53 (69.7%) patients with unilateral disease and 23 (30.3%) with bilateral disease. The average relapse rate was 0.46 ± 0.23/year (range: 0.14–1/year). The details of the demographic and clinical characteristics of middle-aged and older patients are summarized in Table 1. All patients received intravenous methylprednisolone therapy (1 g/day) for three days, followed by oral prednisolone (1 mg/kg/day) for two weeks, in keeping with the recommendations of the ONTT [4].

### 3.2. Visual Acuity

Vision loss was severe at onset, with BCVA < 20/400 in 74 (74.8%) eyes. 49 (72.06%) patients aged ≤ 65 years and 8 (100%) patients aged ≥ 65 years had BCVA < 20/400. At the last follow-up, 30 (44.12%) patients aged ≤ 65 years and 7 (87.5%) patients aged ≥ 65 years had BCVA < 20/400. The initial BCVA of the patients aged < 65 years was significantly better from that of the patients aged ≥ 65 years (*p* = 0.006); however, there was no difference in final BCVA (*p* = 0.740). There was also no significant difference between initial BCVA and final BCVA in patients < 65 or ≥65 years (*p* = 0.076, *p* = 0.200). The detailed comparison is summarized in Table 2.

### 3.3. Immunological Findings

Of the 76 patients, 49 patients had AQP4-Ab test, of which 11 (22.4%) were seropositive and 38 (77.6%) were seronegative. 64 patients had autoimmune antibody screening test, and 21 (21.6%) were abnormal, including antinuclear antibody (ANA), anticardiolipin antibody (ACA), double-stranded DNA (dsDNA), anti-Ro antibody (SS-A), anti-La antibody (SS-B), anti-*β*-glycoprotein antibody, rheumatoid factor (RF), and HLA-B27. The positive rate was not related to AQP4-Ab rate. There was no significant difference between patients < 65 years and ≥65 years.

### 3.4. MR Imaging Findings

Of the 76 patients, 53 patients had orbital MRI at presentation, of which 44 (83.0%) had optic nerve T2 lesions or optic nerve enhancement. 50 patients had head MRI imaging at the onset, 5 (10%) had demyelinating lesions, 8 (16%) had T2 lesions, and 9 (18%) had ischemic foci. The abnormal to normal ratio was not significantly different between patients < 65 years and ≥65 years.

### 3.5. OCT Imaging Findings

**Figure 1:**
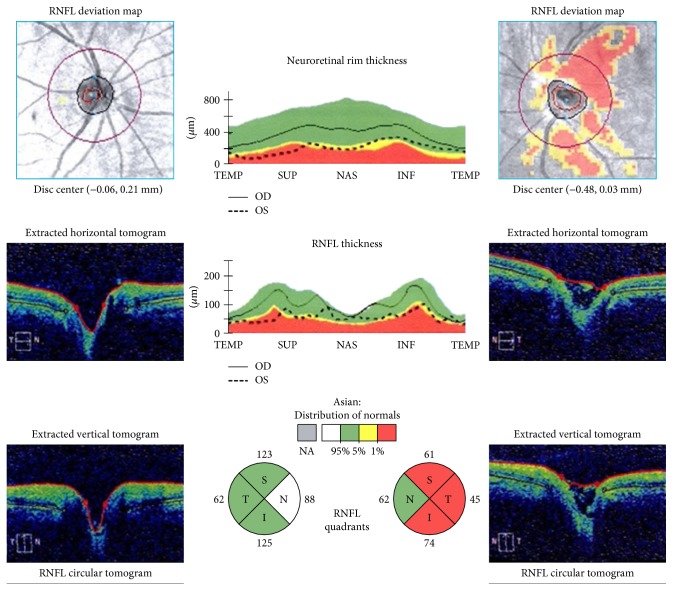
Peripapillary retinal nerve fibre layer (pRNFL) thickness measured by OCT. Temporal pRNFL was significantly reduced in eyes with BCVA < 20/400, (*p* < 0.05).

Of the 76 patients, OCT was performed in 51 patients, with the average time from the onset being 22.75 ± 23.00 months. Of the 51 patients, 45 patients were <65 years and 6 were ≥65 years. The peripapillary retinal nerve fibre layer (pRNFL) thickness (*μ*m) was measured at the final follow-up. The thickness of pRNFL was not significantly different between patients < 65 years and ≥65 years, but the temporal thickness was significantly reduced in those with BCVA < 20/400 compared to ≥20/400 (*p* < 0.05) (Figure 1). Thinning of the pRNFL was also found in those with positive AQP4-Ab (*p* = 0.022).

### 3.6. Correlation of Final BCVA with Other Parameters

The final BCVA was found to be significantly correlated only with the initial BCVA and temporal RNFL thickness (*p* = 0.001, *p* = 0.045). Other variables examined in this patient cohort, such as sex, age, laterality, initial disc appearance, systemic association, orbital MRI findings, head MRI, AQP4-Ab status, and average thickness of pRNFL, were not significantly correlated with the final visual outcomes (Table 3).

### 3.7. Final Diagnosis

At the last follow-up, there are 11 patients diagnosed with NMOSD, 4 with CRION, 1 with MS-ON, 1 with infectious ON, and 15 with autoimmune ON. None of the elderly group developed NMOSD or MS.

## 4. Discussion

Only a few cases of ON in middle-aged or elderly patients have been reported to date. In our study, the principal findings in ON patients ≥45 years were predominantly unilateral disease, severe reduction in visual acuity at presentation, and little recovery at 5-year follow-up.

The ONTT reported good recovery of BCVA in younger patients (18–45 years), with 36% having initial BCVA ≤ 20/200 and 8% with final BCVA ≤ 20/200 [4]. Other studies which included ON patients of all ages also reported better outcomes than our observation. A study of 27 native African patients conducted in Nigeria reported initial BCVA ≤ 20/200 in 60% and only 9% at 12-week follow-up [7]. However, the study only included 1 patient who was older than 50 years.

In the first ON study in older patients reported in 1988, outcomes were worse compared to the above two studies, with 61% having initial BCVA ≤ 20/200 and 17% final BCVA ≤ 20/200 [14].

A recent study, which is conducted in Korea, reported that patients > 50 years recovered well from ON attacks; all affected eyes had final BCVA > 20/40 within two months [15]. However, the small sample size, eight patients in total, may result in increase of the bias. In our study, we divided patients into two subgroups, one middle-aged group (45–65 years) and one elderly group (age ≥ 65 years). In comparison with the above studies, 64% of the middle-aged group and 100% of the elderly group had initial BCVA < 20/200 and 43% of the middle-aged group and 88% of the elderly group had final BCVA < 20/200, showing that visual recovery correlated inversely with age, that is, older patients recovered less vision in this cohort.

The first identified predictor of visual outcomes in this study is initial BCVA. ONTT reported a significant correlation between initial and final BCVA, consistent with our observations [6]. Other predictor in this study is the changes of pRNFL thickness in the temporal. pRNFL thinning manifested within 3 to 6 months after acute ON attack, then remained stable [16]. At 5-year follow-up, we observed particularly thinning in the temporal in patients with final BCVA < 20/400, compared to patients with final BCVA ≥ 20/400. Similar findings have been previously reported in ON patients by Pro et al. [17].

At 5-year follow-up, there were 11 patients diagnosed with NMOSD, 4 with CRION, and 1 with MS-ON. None of the elderly patients developed NMOSD or MS. Despite severe visual loss, elderly patients were less likely to have a diagnosis of NMOSD or MS. ONTT concluded that the risk of developing to MS is heavily dependent upon the MRI findings at onset [6]. For those patients with a normal brain MRI, a 15-year MS risk was 25%. In contrast, those with one or more MRI lesions consistent with demyelination had a 72% risk, with a higher lesion count conferring an even greater risk [18]. It has also been recognized that the risk of developing to NMOSD is higher in patients of Asian origin [19]. This may be associated with the high detection rate of AQP4-Ab in Asia (32.6%) compared to western countries (5.8%) [20, 21].

Myelin oligodendrocyte glycoprotein antibody (MOG-Ab) is also found associating with ON [22–24]. It was initially reported in a study of paediatric demyelinating disease and further reported in a series of related disorders: NMOSD, acute disseminated encephalomyelitis (ADEM), cortical encephalitis, Hashimoto's encephalopathy, epilepsy, LETM, recurrent ON, and MS [25–27]. However, because this is a retrospective study, MOG antibody test was not available at the time. The strength of our study is that we carefully excluded the diagnosis of NAION in this cohort. NAION constitutes one of the major causes of blindness or seriously impaired vision among the middle-aged and elderly population [28]. Previous study on patients older than 50 years concluded that 6.25 patients per 100000 inhabitants were affected with NAION per year in Beijing [29]. NAION and ON patients share many symptoms and signs [30]. In this study, we did not only exclude patients with the risk factors for NAION, but also used MRI and FFA to further confirm the diagnosis, in order to obtain the uniform sample of the first episode of optic neuritis.

## 5. Conclusions

In summary, ON in the older population is less likely to develop to NMOSD or MS, especially in elderly patients (≥65 years), even though patients tend to present with more severe visual loss and poor recovery following classic steroid treatment. Further prospective studies are needed to confirm our observations and find an optimal treatment strategy for this population.

## Figures and Tables

**Table 1:** Baseline characteristics of middle-aged and older patients.

Baseline characteristics		Frequency	Middle-aged group (age < 65 years)	Elderly group (age ≥ 65 years)	*p* value
Total patients		76	68	8	—
Age at onset (years)	Average	55.53 ± 8.29 (45–83)	53.28 ± 5.05 (45–65)	74.75 ± 4.56 (72–83)	NS
Gender *n* (%)	Male	44 (57.9)	30 (44.1)	2 (25)	NS
	Female	32 (42.1)	38 (55.9)	6 (75)	
Laterality, *n* (%)	Unilateral	53 (69.7)	49 (72.1)	4 (50)	NS
	Bilateral	23 (30.3)	19 (27.9)	4 (50)	
Pain, *n* (%)	Present	25 (53.2)	22 (50.0)	3 (100)	NS
	Absent	22 (46.8)	22 (50.0)	0 (0)	
Initial disc appearance, *n* (%)	Present	30 (40.5)	29 (50.0)	1 (87.5)	NS
	Absent	44 (59.5)	37 (50.0)	7 (12.5)	
Autoimmune antibodies, *n* (%)	Normal	43 (67.2)	38 (67.9.0)	5 (67.2)	NS
	Abnormal	21 (21.8)	18 (32.1)	3 (32.8)	
AQP4 antibody, *n* (%)	Seropositive	10 (20.4)	10 (22.2)	0 (0)	NS
	Seronegative	39 (79.6)	35 (77.8)	4 (100.0)	
CSF study, *n* (%)	Normal	23 (50.0)	23 (51.1)	0 (0)	NS
	Abnormal	23 (50.0)	22 (48.9)	1 (100.0)	
Orbit MRI, *n* (%)	Normal	9 (17.0)	7 (14.9)	2 (33.3)	NS
	Abnormal	44 (83.0)	40 (85.1)	4 (66.7)	
Head MRI, *n* (%)	Normal	26 (52.0)	25 (55.6)	1 (20.0)	NS
	Abnormal	24 (48.0)	20 (44.4)	4 (80.0)	
Relapse rate (N/Y)	Average	0.46 ± 0.23 (0.14–1)	0.46 ± 0.23	0.44 ± 0.18	NS

CSF: cerebrospinal fluid; AQP4-Ab: anti-aquaporin-4 antibody; MRI: magnetic resonance imaging.

**Table 2:** Factors for visual prognosis in middle-aged and elderly patients with optic neuritis.

Factors		Number of patients with final VA < 20/400	Number of patients with final VA ≥ 20/400	*p* value
Initial BCVA	>20/400	3	16	0.001^∗^
	<20/400	35	22	
Sex	Male	15	17	NS
	Female	23	21	
Age	<65	31	37	NS
	≥65	7	1	
Laterality	Unilateral	24	29	NS
	Bilateral	14	9	
Initial disc appearance	Normal	23	21	NS
	Swollen	14	16	
Systemic association	Normal	20	23	NS
	Abnormal	10	11	
CSF	Normal	10	12	NS
	Abnormal	13	11	
Serum AQP4-Ab status	Seropositive	7	3	NS
	Seronegative	17	22	
Orbital MRI	Normal	4	5	NS
	Abnormal	22	22	
Brain MRI	Normal	11	15	NS
	Abnormal	13	11	
pRNLF	Average	64.00 ± 12.91	68.58 ± 18.70	NS
	Superior	69.52 ± 20.72	79.23 ± 32.63	NS
	Nasal	59.60 ± 9.39	60.35 ± 10.00	NS
	Inferior	72.88 ± 23.53	83.88 ± 30.37	NS
	Temporary	50.85 ± 15.81	52.92 ± 10.15	0.045^∗^

BCVA: best corrected visual acuity; CF: count finger; CSF: cerebrospinal fluid; AQP4-Ab: anti-aquaporin-4 antibody; MRI: magnetic resonance imaging; pRNLF: peripapillary retinal nerve fibre layer, NS: nonsignificant; ^∗^*p* < 0.05.

**Table 3:** Visual outcomes Of Chinese middle-age and elder patients with optic neuritis.

Visual outcomes (worse eye at onset if bilateral)	Initial BCVA		Final BCVA	
	Age < 65 years	Age ≥ 65 years	Age < 65 years	Age ≥ 65 years
NLP ≤ BCVA < CF	31 (45.6)	8 (100)	16 (23.5)	2 (25.0)
CF ≤ BCVA < 20/400	18 (26.5)	0	14 (20.6)	5 (62.5)
20/400 ≤ BCVA < 20/66	14 (20.6)	0	17 (25.0)	0
20/66 ≤ BCVA < 20/20	3 (4.4)	0	17 (25.0)	1 (12.5)
BCVA ≥ 20/20	2 (2.9)	0	4 (5.9)	0
Total	68	8	68	8
Average LogMar BCVA	1.94 ± 0.86	2.63 ± 0.30	1.28 ± 0.98	1.72 ± 0.83
*p* value	0.006^∗^		NS	

NLP: light perception; LP: light perception; BCVA: best corrected visual activity; ^∗^*p* < 0.05.
